# Automatic imitation in a rich social context with virtual characters

**DOI:** 10.3389/fpsyg.2015.00790

**Published:** 2015-06-09

**Authors:** Xueni Pan, Antonia F. de C. Hamilton

**Affiliations:** Institute of Cognitive Neuroscience, University College LondonLondon, UK

**Keywords:** automatic imitation, virtual reality, social facilitation effect, action sequencing, virtual characters

## Abstract

It has been well established that people respond faster when they perform an action that is congruent with an observed action than when they respond with an incongruent action. Here we propose a new method of using interactive Virtual Characters (VCs) to test if social congruency effects can be obtained in a richer social context with sequential hand-arm actions. Two separate experiments were conducted, exploring if it is feasible to measure spatial congruency (Experiment 1) and anatomical congruency (Experiment 2) in response to a VC, compared to the same action sequence indicated by three virtual balls. In Experiment 1, we found a robust spatial congruency effect for both VC and virtual balls, modulated by a social facilitation effect for participants who felt the VC was human. In Experiment 2 which allowed for anatomical congruency, a form by congruency interaction provided evidence that participants automatically imitate the actions of the VC but do not imitate the balls. Our method and results build a bridge between studies using minimal stimuli in automatic interaction and studies of mimicry in a rich social interaction, and open new research venue for future research in the area of automatic imitation with a more ecologically valid social interaction.

## Introduction

Mapping one's own body and actions to the body and actions of others is a core mechanism for social cognition. Multiple studies have shown that people respond faster and more accurately when they have the chance to perform an action that is congruent with an observed action than when they respond with an incongruent action (Brass et al., 2000; Stürmer et al., 2000; Cook and Bird, 2011). However, the majority of these studies use very minimal stimuli (e.g., an image of an isolated hand). Here we test if social congruency effects can be obtained in a richer social context with sequential hand-arm actions performed by a virtual character (VC). We further explore if these effects are modulated by spatial congruency or by anatomical congruency. First, we review past studies on social action congruency effects, and on the use of VCs to explore social interaction.

### Social congruency effects

Automatic imitation occurs when a participant responds faster in an imitative context than in a matched, non-imitative context, and provides a robust measure of how easily a participant maps actions between self and other. Two early papers developed automatic imitation paradigms which have been widely used in social neuroscience (Brass et al., 2000; Stürmer et al., 2000). In Brass et al.'s study, participants were instructed to respond to a symbolic number cue (1 or 2) while ignoring an irrelevant finger movement in the background. Reaction times were faster when the irrelevant finger movement on the screen was congruent with the participant's response than when it was not (Brass et al., 2000). In Strümer's study, participants were instructed to make a pre-specified movement (either hand-opening or hand-closing) as soon as they saw a hand movement on the screen. Responses were faster when the instructed response was congruent with the stimulus than when it was incongruent (Stürmer et al., 2000).

A key requirement for an automatic imitation effect is that it is driven by a precise mapping between one's own body and the body of the actor, and not purely by the spatial locations of items in the field of view. It has been shown that Brass et al.'s finger-movement task and Stürmer et al.'s hand-opening task both measure a true imitation effect because both are robust to changes in the orientation of the stimuli (Heyes et al., 2005; Bertenthal et al., 2006; Cook and Bird, 2011). For instance in Heyes et al.'s version of the hand-opening task, the stimulus hand was vertically aligned and the responding hand (participant' hand) was horizontally aligned (Heyes et al., 2005) so congruent responses are anatomically but not spatially matched. In Bertenthal et al.'s version of the finger-tapping task, participants were instructed to perform finger-tapping toward both left hand and right hand as stimuli (Bertenthal et al., 2006). They found evidence for both spatial compatibility and automatic imitation effects, with the latter decreasing over the course of each individual experimental block. This suggested that both effect exist independently.

The automatic imitation effect can be modulated by the form of the actor: several studies have found that the effect is stronger for human than non-human hands, however it is still present for the latter (Press et al., 2005; Longo et al., 2008; Longo and Bertenthal, 2009; Liepelt and Brass, 2010). In Press et al.'s study, using the hand-opening task, participants were presented with both human and robot hands (Press et al., 2005). It was found that there was a congruency effect with both forms of hands, as well as an interaction between stimulus form and congruency, indicating that the congruency effect was greater with the human hand (27.9 ms) than with the robotic hand (8.8 ms). Similar results were also obtained in Liepelt and Brass's study using the finger tapping task, where participants were primed to believe that the video of a hand (covered with a glove) was either a real human hand or a wooden hand (Liepelt and Brass, 2010). Although the actual video stimuli were identical, participants in the wooden-hand group showed a reduced congruency effect as result of priming. In Longo, Kosobud, and Bertenthal's study, participants were presented with computer-generated realistic looking hand, animated with either biomechanically possible or impossible movements (Longo et al., 2008). The compatibility effect was present in both automatic (Experiment 1) and spatial (Experiment 3) imitation, and the results were similar regardless of the type of stimuli (biomechanically possible or impossible). However, in their second experiment, when participants were explicitly informed about the movements before the experiment, the compatibility effect disappeared with the biomechanically impossible movements (only automatic imitation was tested in this experiment). A follow up study found that automatic imitation of a virtual hand was reduced - but not eliminated—when participants were informed that they were going to see a virtual hand (Longo and Bertenthal, 2009). Overall, these studies have shown that automatic imitation can be obtained for human, mechanical and computer-generated hands, with the magnitude of the effect dependent on participant's beliefs about the hand.

### Richer contexts

One limitation of current studies of automatic imitation is that they mostly used isolated hand stimuli and limited contexts. A few studies have explored larger social contexts by adding faces to moving hands (Wang et al., 2010; Grecucci et al., 2013). Grecucci et al. displayed faces with either neutral or negative emotion before each stimulus, and instructed both ASD children and health controls to perform finger-tapping presented with finger-tapping (compatible) or finger-lifting (incompatible) stimuli (Grecucci et al., 2013). It was found that both ASD and control groups had a compatibility effect, and that the control group had a significant faster response toward the stimuli following the display of negative faces, whereas this effect was not present with the ASD group. Wang, Newport, and Hamilton displayed faces with direct or averted gaze before a hand-opening/closing stimulus and measured congruency effects (Wang et al., 2010). They found that participants were faster at the congruent trials with the direct gaze than with the averted gaze in a hand-opening task, indicating that direct gaze enhances automatic imitation.

Others have added social priming before a hand action imitation task (Leighton et al., 2010; Wang and Hamilton, 2013). Using a scrambled-sentence paradigm, Leighton et al. found that pro-social priming elicited a larger automatic imitation effect in a hand-opening task, whereas anti-social priming elicited a reduced automatic imitation effect (Leighton et al., 2010). Wang and Hamilton further argued that such a pro- or anti-social priming effect is modulated by self-relatedness. They found that first-person prosocial and third-person antisocial primes both increased automatic imitation (Wang and Hamilton, 2013). A full review of the many factors modulating automatic imitation can be found in Heyes (2011) and Wang and Hamilton (2012).

The aim of the current paper was to test if automatic imitation effects can be obtained robustly in an even richer context, where participants perform actions in front of a life-size VC. VCs have been valuable in the study of human social interaction in various ways. Early studies in this area used a virtual ball tossing game with simple VCs to investigate perspective taking (David et al., 2006) and social exclusion (Eisenberger et al., 2003). Other studies use expressive VCs to study the social function of gaze (Georgescu et al., 2013), blushing (Pan et al., 2008), and mimicry (Bailenson and Yee, 2005). More recently, photo-realistic looking VCs animated with motion-captured data were used in studies of joint action (Sacheli et al., 2015), embodiment (Kilteni et al., 2013), and personality (Pan et al., 2015). The high level realism of both appearance and behavior in these studies provided a key element in achieving ecological validity.

In the present study, we used VCs to prime the performance of action sequences and test if automatic imitation can be obtained for sequential actions in a rich social context. On each trial, the VC performed a sequence of three actions, and then the participant was instructed to perform a sequence which could be congruent or incongruent with the actions of the character. As a control condition, participants saw three balls which touch the same goal locations as the VC, without any human form or biological motion. We can identify three possible effects. First, we could find a main effect of congruency, with faster responses following congruent actions. Different configurations of the action goals can allow us to distinguish between spatial congruency and anatomical congruency (see below). Second, we could find a main effect of actor form, whereby participants are faster to response when a VC is present. This would be a social facilitation effect, where responses are faster when participants are in the presence of a real (or virtual) human (Bond and Titus, 1983). Finally, we could find a congruency by form interaction, where form could be a virtual human or a non-human object (a moving ball). This is the signature of automatic imitation, because it indicates that participants are faster on congruent trials only when the actions are performed by a VC with a comparable body shape to the participant, and not when the same goals are indicated by a non-human object. Spatial effects can be ruled out.

There are two ways in which the actions of the participant could be congruent with the actions of the avatar. They could be directed to the same location in space (spatial congruency), or they could use the same arm movements (anatomical congruency). We test the former in Experiment 1, and the latter in Experiment 2. Based on previous findings that automatic imitation for simple finger movements is driven by anatomical effects, we predict that when movements are spatially (but not anatomically) congruent, we would find only a main effect of congruency (Experiment 1). We further predict that when movements are anatomically matched between participant and avatar, we would find a form by congruency interaction, indicating a true automatic imitation effect (Experiment 2).

## Experiment 1—spatial congruency

### Participants

A total of 22 participants were recruited from the ICN Subject Database (14 females; mean age = 22.5 years; *SD* = ±4.3 years). All were right-handed (by self-report), had normal or corrected-to-normal vision, and were naïve to the purpose of the study. They received payment at the end of the study. The study was approved by the UCL graduate school ethics committee.

### Materials

**Figure 1:**
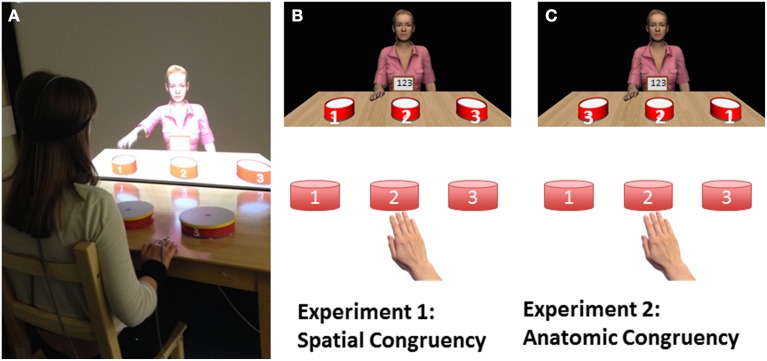
**(A)** A photo of the study in progress. The participant has a Polhemus magnetic marker on her head and right hand to track her movements. **(B)** Configuration for Experiment 1. The drum numbering and the participant's responses are spatially congruent between participant and VC. **(C)** Configuration for Experiment 2. The drum numbering and responses are anatomically congruent between participant and VC.

The experiments were conducted in our lab where VR graphics were displayed in 2D on a 90 cm × 160 cm projector screen. As shown in Figure 1A, the lab was prepared with a wooden stool in front of a wooden table with three plastic toy drums on top. Immediately beyond the table was a large projector screen, where the participant could see the virtual world. The virtual environment was modeled to match the real world with a virtual wooden table which looks like an extension of the real one, and three matching virtual drums modeled in 3D Studio Max (Autodesk). The drums on the desk and the drums in the virtual world were numbered 1, 2, or 3 as illustrated in Figure 1B. A female VC (Jessie) sat behind the virtual wooden table, facing the participant. Jessie was animated with pre-recorded motion captured data and was controlled by the VR application in real time. In front of Jessie the participant could see a virtual iPad, where the participant received instructions.

Jessie's motion was obtained by motion-capturing a single female actor performing the same task as participants at the same desk. The actor had four Polhemus Liberty magnetic motion trackers, placed on her head, chest, right hand side elbow, and middle finger. The Polhemus data was streamed into a machine running Motionbuilder (through the Polhemus plug-in for Motionbuilder), which produces character animation after a small calibration session. Unlike from optical motion capture system, the magnetic trackers used here give both position and orientation data and therefore four trackers were enough to produce high quality human-like animation for our setting (upper body with one arm movement). Animation data was saved while the actor performed all possible sequences of taps on the three drums, instructed by number cues. The animation files were stored the Cal3D format and applied to Jessie within our interactive VR application developed in Vizard (WorldViz Inc,).

### Experimental design

A 2 × 2 within participants design was used and the two factors were form (Jessie or balls) and congruency (congruent or incongruent). The number cue, displayed on the virtual iPad during the training and experiment session, consisted of a sequence of three numbers with all possible combinations of 1, 2, and 3, excluding only “1-1-1,” “2-2-2,” and “3-3-3,” This gives 24 possible combinations. Each participant completed 6 blocks (three Jessie, three balls, alternatively): half of them had Jessie as their first block and the other half the balls. Each block consists of 48 trials: 24 congruent trials and 24 incongruent, displayed in random order. Each block could last between 4 and 7 min depending on participants' speed, and the full set of six blocks could be completed in less than 30 min.

### Procedure

On arrival at the lab, each participant was introduced to the VR setup and completed the consent form. Two Polhemus motion-tracking markers were fitted to the participant's right index finger and forehead with medical tape and a headband. The participant then completed a 5-min calibration and training session for the drum tapping. They were instructed to tap each drum in order as soon as they saw the number cue on the virtual iPad, and should then return to the rest position. Participants practiced this for at least 5 successive correct trials before moving on to the main experiment.

**Figure 2:**
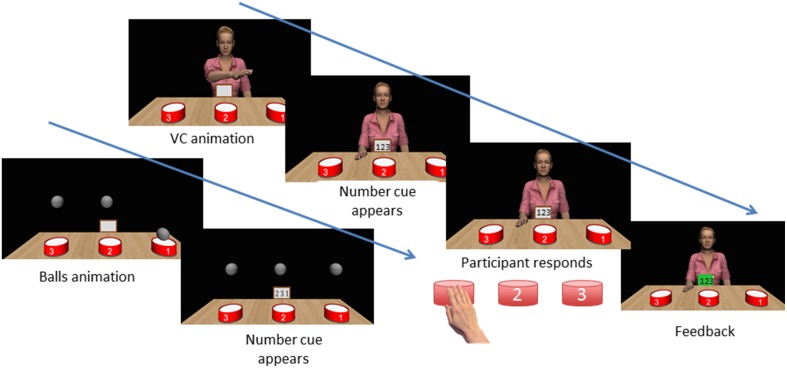
**Sequence of events in a trial**. The VC completes a sequence of action. A number cue appears on the “virtual ipad” in front of the VC. The participant taps each drum in the sequence as fast as possible. The participant receives feedback on correct/erroneous sequences. Note that at all points except the VC animation, the VC's head and gaze track the participant's head to give a feeling of actively being watched.

For the main experiment, participants were instructed that they would perform the same drum tapping task, taking turns with Jessie or with some balls. For each trial, the participant first saw either Jessie or the balls tap a three-beat sequence (e.g., 2-1-3) which lasted approximately 3 s. A drum sound effect played at each point when Jessie or the balls hit each drum. Then the virtual iPad provided a number cue instructing the participant to perform a three-beat sequence (Figure 2). Unbeknownst to the participant, these sequences can be congruent to the action of the VC (e.g., “2-1-3”) or incongruent (e.g., “3-1-1”). In the congruent trials the VC would tap the same spatial locations as the participant i.e., both the physical and virtual drum “1” was at left-hand side of the participant (spatial congruency). Both the participant and Jessie used their right hand, so a reach to drum 1 was a contralateral movement for the participant but an ipsilateral movement for Jessie. This means that the spatially congruent actions were not anatomically congruent. The incongruent animations were designed to be incongruent both anatomically and spatially. For instance, in an incongruent trial, when the participant was cued to tap “2-1-3,” the animation was neither “2-1-3” nor “2-3-1.”

During the participant's response period, Jessie would “actively watch” the participant. This means that Jessie's head rotation (left/right, up/down) were programmed so that Jessie was always looking at the participant—if the participant moved slightly left, Jessie looked slightly to the left. This was implemented using the “lookAt” function in Vizard, setting Jessie's head to orient toward an invisible virtual object whose position was tied to the motion tracker on the participant's head during participant's response period, and was tied to the position of the middle virtual drum during Jessie's tapping session. For transitions between the response period and Jessie's drumming, the position of the virtual object was updated by linearly interpolating between the two possible positions over 0.5 s. This ensured that Jessie produced smooth, realistic and socially engaging head movements over the whole study. Participants did not explicitly notice that Jessie was actively watching them during the response phase, but we found it increased the feeling of social engagement and realism.

The motion tracking data collected from the participant's hand was used to monitor performance online. The times when participants touched each drum were defined by the Vizard function “vizproximity” set to detect when the hand marker moved within approximately 1 cm of the center of the drum. The drum-tapping sound effect was played as the participant tapped each drum. Any errors (tapping the wrong drum) resulted in the virtual iPad turning red and a harsh beep sound. When a trial was correctly completed, the virtual iPad turned green. The end of a trial was triggered when the participant's hand returned to the resting location, and the next trial began immediately.

Blocks with ball stimuli were matched in all features, except that Jessie was not present and instead the participant sees three balls suspended above the three drums. To tap a sequence, one ball at a time moved downwards with a constant velocity, tapped the drum and returned to its place. This was implemented using the “moveTo” build-in function in Vizard (Figure 2 and Video 1 from Supplement Material).

After participants completed all six blocks of the task (three blocks with Jessi and three blocks with balls), they filled in an online questionnaire concerning their subjective evaluation of the experience and of Jessie's personality (see Data Sheet 1 in Supplementary Material). Participants gave their subjective social evaluation (*SE*) toward the VC through two questionnaires (co-presence and personal trait evaluation). These questions were adapted from previous Virtual Reality studies involving human-VC interactions (Pan et al., 2008, 2015), and here a Likert Scale of 1–7 (1: not at all; 7: very much so) was used. The average score across all 10 questions was used as a covariate in the data analysis. Finally, participants were debriefed and were paid for their time.

### Data analysis

**Figure 3:**
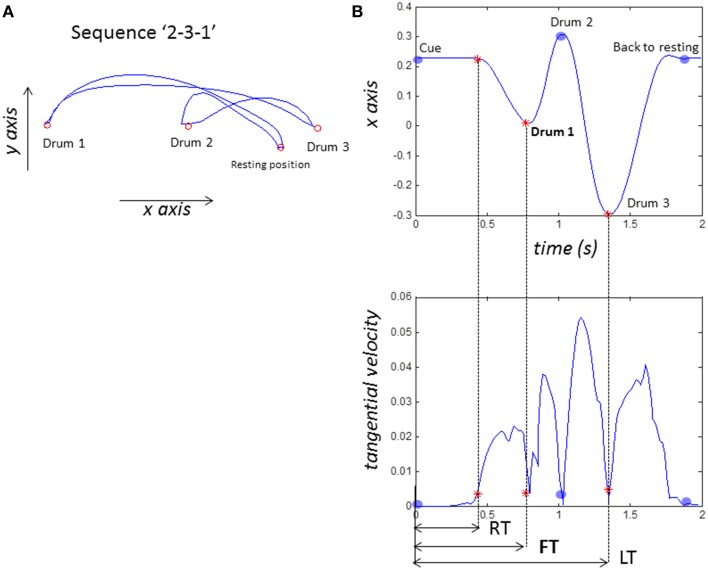
**Sample data from one participant performing the sequence “2-3-1.” (A)** sample plot of hand kinematics **(B)** definitions of timings. X-axis is left and right, tangential velocity is calculated off line with Matlab. RT (reaction time) or movement onset is defined as when tangential velocity >0.0035 m/s. FT (first action time) is when participant taps their first drum (drum 2 in this case). LT (last action time) is when they taps their last drum (drum 1).

Each participant completed 288 trials equally spread across the following four conditions, with 72 trials in each condition: *congruent-balls* (*CB*), *incongruent-balls* (*IB*), *congruent-VC* (*CV*), and *incongruent-VC* (*IV*). Two.csv files were produced in real-time with our Vizard application: (1) *event file* contains the time and type of events (e.g., number cue display, participant taps the first drum, and participants' action was correct or incorrect) (2) *tracking file* contains time and motion captured data (position and rotation). In our analysis only position data was used. The following features were extracted for each trial (see Figure 3):

- Reaction time (*RT*): The time from the onset of the number cue to the first hand movement, was calculated offline with Matlab. RT was defined as the point when tangential velocity of the finger marker surpassed 0.0035 m/s.
- First drum time (*FT*): The time from the onset of the number cue to the time when the participant tapped the first drum of the sequence of three. This is our primary outcome measure and was recorded in real-time with our Vizard application.
- Last drum time (*LT*): The time from the onset of the number cue to the time when the participant tapped their last drum in the sequence of three; this was recorded in real-time with our Vizard application.
- Errors (*ER*): correct or error in the response.

Each of the four features was averaged at condition level for each participant. For *RT, FT*, and *LT*, incorrect trials, or trials where *FT* (our primary measurement) is more than two standard deviations from the mean were excluded from the analysis (4.2%). Data for each of the four features was input to a repeated-measures ANOVA, both with and without mean SE scores as a covariate. Our primary outcome measure was the time to touch the first drum (FT) and we report this measure in the text and tables. Other measures (*RT, LT*, and *ER*) are presented in Tables 1–4 only, for completeness.

**Table 1:** **Experiment 1: repeated measure ANOVA (*n* = 22)**.

	**Measure**	***F***	***P***	***Partial* η^2^**
RT	cong	10.268	0.004	0.328
FT	cong	25.623	0.000	0.550
LT	cong	13.228	0.002	0.386
ER	cong	6.720	0.017	0.242

**Table 2:** **Experiment 1: Repeated measure ANOVA with SE as covariance (*n* = 22)**.

	**Measure**	***F***	***P***	***Partial* η^2^**
RT	form	5.510	0.029	0.216
	form^*^SE	10.251	0.004	0.339
FT	form	8.954	0.007^a^	0.309
	form^*^SE	13.665	0.001^a^	0.406
	cong	5.928	0.024	0.229
LT	form	5.558	0.029	0.217
	form^*^SE	7.355	0.013	0.269
	cong	12.913	0.002^a^	0.392
	cong^*^SE	5.567	0.029	0.218

a *The effect is still preserved (p < 0.05) after we remove a potential outlier with SE > 6*.

**Table 3:** **Experiment 2: repeated measure ANOVA (*n* = 32)**.

	**Measure**	***F***	***P***	***Partial* η^2^**
RT	form	9.349	0.005	0.232
	cong	11.790	0.002	0.276
FT	form	14.930	0.001	0.325
	cong	13.018	0.001	0.296
	form^*^cong	5.246	0.029	0.145
LT	form	4.281	0.047	0.121
	cong	7.437	0.010	0.193

**Table 4:** **Experiment 2: repeated measure ANOVA with SE as a covariate (*n* = 32)**.

	**Measure**	***F***	***P***	***Partial* η^2^**
RT	cong	5.290	0.029	0.150
FT	cong	8.325	0.007	0.217
LT	cong	11.673	0.002	0.280
	cong^*^SE	6.270	0.018	0.173
ER	cong	5.294	0.029	0.150
	cong^*^SE	6.921	0.013	0.187

### Results

The mean error rate was 1.2%. SE scores had a mean of 3.45 (SD 1.29).

**Figure 4:**
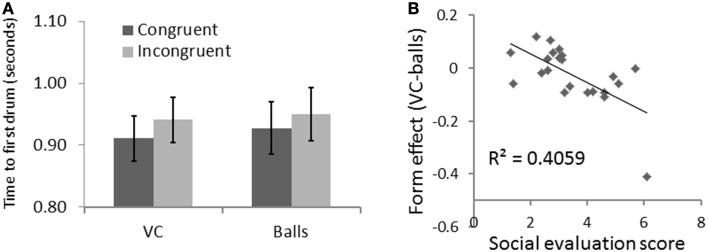
**Results for Experiment 1 with SE as a covariate. (A)** Time to reach the first drum was faster for VC than for balls. **(B)** This effect correlated with the social evaluation score.

A repeated measure ANOVA revealed a congruency effect for first *drum time* [*F*_(1, 21)_ = 25.62, *p* < 0.001, η^2^ = 0.550] indicating that participants were faster in the congruent trials than the incongruent trials. There was no form effect or interaction effect. See Figure 4A.

A repeated measure ANOVA taking participants' *SE* score as a covariate revealed a similar congruency effect [*F*_(1, 20)_ = 5.93, *p* = 0.024, η^2^ = 0.229]; a form effect [*F*_(1, 20)_ = 8.95, *p* = 0.007, η^2^ = 0.309], indicating that participants reached the first drum faster with the VC than drums; and form-SE interaction [*F*_(1, 20)_ = 13.67, *p* = 0.001, η^2^ = 0.406]. To explore the direction of this effect, we calculated a form effect for each participant as the first-drum-time for the VC minus the first-drum-time for the balls. As shown in Figure 4B, the form effect was negatively correlated with SE (*R* = −0.637, *R*^2^ = 0.406, *p* = 0.001). This means that the more a participant felt socially connected to the VC, the quicker they reacted to the VC compared to the balls.

### Discussion

Our results from Experiment 1 show a main effect of congruency, but no other effect was significant. This can be accounted for by a purely spatial effect, where participants were faster to respond to a particular sequence when they had just viewed a sequence directed toward the same drum locations. This is in line with our prediction that spatial congruency between the participant's drums and the VCs drums should lead to purely spatial effects.

Furthermore, when taking into account participant's reported level of SE of the VC, we found a main effect of form and a form-SE interaction. These results suggest that a social facilitation effect can be obtained using our VC, where participants are faster to respond to a human-like VC than to non-human balls. This is compatible with previous reports of social facilitation to computer generated figures (Hoyt et al., 2003; Zanbaka et al., 2007).

## Experiment 2: anatomic congruency

### Participants

A total number of 32 participants (24 females; mean age = 23.1 years; *SD* = ±3.73 years) attended Experiment 2. All were right-handed, had normal or corrected-to-normal vision, and were naïve to the purpose of the study. They received payment at the end of the study. The study was approved by the UCL graduate school ethics committee.

### Experimental design

The experimental design and trial arrangement closely matched Experiment 1. As shown in Figure 1C, the only difference was that the virtual drums were displayed in the opposite order as compared to Experiment 1. This means that the participant reaches contralaterally to drum 1, and the VC also reaches contrallateraly to drum 1. These movements are anatomically congruent but not spatially congruent. All trials for this experiment were defined in terms of anatomical congruency (not spatial congruency). Note that there was no need to record new animation clips, because the animation clip of the VC playing “2-1-3” in Experiment 1 was the same as that of “2-3-1” in Experiment 2. Instructions, trial structure and trial numbers were identical to Experiment 1. Participant filled the same SE questionnaire and an SE score was calculated. As before, we report analysis over our main measurement (*FT*) in the text and figures, and present data from all measurements (*RT, FT, LT*, and *ER*) in Tables 3, 4.

### Results and discussion

The mean error rate was 1.5%. Again, for *RT, FT*, and *LT*, incorrect trials, or trials where *FT* (our primary measurement) is more than two standard deviations from the mean were excluded from the analysis (4.0%). SE scores had a mean of 2.88 (SD 1.16). A *t*-test directly comparing SE scores in Experiment 1 vs. Experiment 2 did not show a significant different (*p* = 0.095). We speculate that in Experiment 1 it was easier to map spatially between the participant's action and the VC's action on congruent trials, leading the participant to feel similar to the VC. In contrast, in Experiment 2 the participant had to mentally rotate his/her body to the location of the VC to create a strong self-other correspondence on congruent trials. This difference in the ease of self-other mapping between the studies might lead to a trend toward a difference in SE scores. This parallels previous reports that mimicry (mirroring) enhances liking even in VCs (Bailenson and Yee, 2005; Gratch et al., 2007).

**Figure 5:**
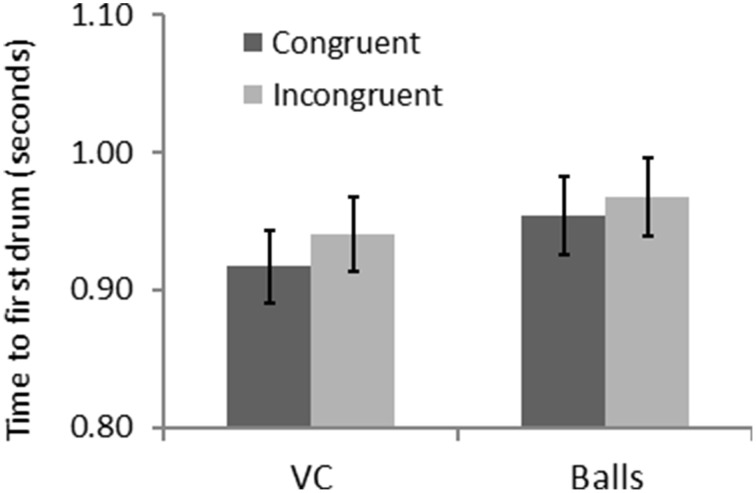
**Results for Experiment 2—congruency and form interact for time to first drum**.

A repeated measure ANOVA revealed a significant effect of form [*F*_(1, 31)_ = 14.93, *p* = 0.001, η^2^ = 0.325] and congruency [*F*_(1, 31)_ = 13.02, *p* = 0.001, η^2^ = 0.296] for *FT*. Figure 5 shows that participants were faster in the congruent trials, and that they were also quicker with the VC than the balls. There was also an interaction of form and congruency [*F*_(1, 31)_ = 5.25, *p* = 0.029, η^2^ = 0.145] for FT: the congruency effect is bigger with the VC than the balls. This is consistent with an automatic imitation effect.

A repeated measure ANOVA taking participants' *SE* score as a covariate preserved the congruency effect for FT [*F*_(1, 30)_ = 8.33, *p* = 0.007, η^2^ = 0.217] but the form effect and interaction were no longer present. There were also no effects of SE or interactions with SE, suggesting that SE does not add explanatory value to our model but rather reduces power. Thus, we focus our discussion on the basic model without additional covariates.

To summarize, Experiment 2 revealed a congruency effect with faster responses to anatomically congruent trials, and a form effect, suggesting that participants were faster with the VCs as compared to the balls. More importantly, the reliable form by congruency interaction indicates that participant's automatically imitate the actions of the VC but do not imitate the balls.

## General discussion

In this study, we test if automatic imitation can be obtained in a rich social context with a VC performing sequential actions. We find that spatial congruency effects can be obtained in a context where the virtual drums spatially match the participant's drums (Experiment 1) while automatic imitation can be obtained in a context where the VC's movements anatomically match the participant's movements. These results confirm that automatic imitation can be studied in a richer social context with sequential actions. We consider first the general implications of our novel task and then the specific spatial and anatomical versions of the task.

### Measuring social congruency with a drumming task

In this study we developed a new drumming task for measuring and manipulating automatic imitation. Our task differs from previous tasks (Brass et al., 2000; Stürmer et al., 2000) in at least three ways—it involves sequential actions, it involves goal-directed actions and it is embedded in a rich social context. Sequential actions are an advantage because there are more action options available. This means that our control (incongruent) condition in the sequence task with 3 drums can have neither anatomical nor spatial congruency, thereby providing a better baseline. However, there is also a limitation that automatic imitation can only occur if participants remember the three item sequence from demonstration to the trial. Previous studies of automatic imitation have used simple, single actions (Brass et al., 2000; Stürmer et al., 2000), and it could be argued that the present study does not tap automatic imitation because the action sequences are too complex.

However, there are several reasons to believe that sequential actions can also drive imitation without awareness. Heyes' influential associative sequence learning model of imitation includes action sequences as well as simple stimulus-response associations (Brass and Heyes, 2005). Careful video analysis of natural human behavior also shows copying of action sequences (Grammer et al., 1998). Neuroimaging studies suggest that action sequences and simple actions are stored in a hierarchical format across the cortex (Hamilton and Grafton, 2007). The present study also provides an opportunity to test the hypothesis that action sequences can drive automatic imitation in the same way as simple actions, and provides a positive answer.

A possible limitation of the present study is that verbal encoding of the sequences (both the VCs sequence and the participants sequence) could interfere with the automatic imitation effect. However, verbal encoding would not lead to an advantage in performance that is specific to the human—congruent condition. The fact that we are able to demonstrate an automatic imitation effect (form X congruency interaction in Experiment 2) despite these limitations demonstrates that observing an action can have a robust and enduring effect on subsequent performance. Future versions of our task may use color cues or other symbols to reduce the likelihood that participants verbally encode the number cues.

Unlike previous tasks, our drumming task is goal directed and each action leads to a noticeable effect (drum sound). This contrasts with the finger-lifting (Brass et al., 2000) and hand-opening tasks (Stürmer et al., 2000) which are not directed toward a particular object. Automatic imitation has also been shown in finger-tapping tasks (Wang and Hamilton, 2013), suggesting that the absence of a goal is not essential for this effect. The present data adds weight to this conclusion, suggesting that even sequential goal-directed actions can lead to an imitation effect. This is also consistent with data showing imitation of kinematic features of sequential pointing actions (Wild et al., 2010), and point to the generality of imitative behavior.

Finally, our new paradigm allows us to study automatic imitation in a very rich social context with an increase in social and ecological validity. It is socially plausible that sometimes you are required to take turns with other person to play drums. The set up of the study could be interpreted as a joint-action, where “two or more individuals coordinate their actions in space and time to bring about a change in the environment” (Sebanz et al., 2006). Similar actions also occur in the context of music (Keller, 2008). This paradigm can therefore offer more direct insights in interpreting automatic imitation or mimicry in everyday social activities and joint actions (Grammer et al., 1998), and provide a bridge between minimal automatic imitation tasks and real-world social psychology mimicry tasks.

To achieve a high level of ecological validity while retaining experimental control, we make use of virtual reality technology to create realistic and interactive VCs. Our experiment was conducted on a large projector screen with life-sized VCs sitting right in front of participants, and that our Virtual Environment was implemented to look like an extension of our real lab. This is key to allowing real-life like social interaction experience and enables participants' automatic social responses. Slater (Slater, 2009) proposed that the two orthogonal components contributing to participants' realistic response in Virtual Reality are *Place Illusion* and *Plausibility Illusion*. In our study, the *Place illusion* was achieved by matching the virtual world to the real world in physical setting the sizes of objects and people, such that participants could believe they were looking through a window into a virtual world. The *Plausibility Illusion* was achieved via realism and interactivity of the VC. Here realism is generated not only by using photo-realistic VC but also by using motion-captured data to animate its movement. Interactivity is achieved by ensuring that VC looks toward the participant during each response period, and that she reacts toward the participants' movement, always waiting for the participant to finish their trial before starting her own. The interactive behavior of our VC, though subtle, is a very important aspect which provides a feeling of social contingency between the participant and VC. The fact that participants' own action and movement could bring about a change in the behavior of another “person” make the whole experience more social and plausible. This is a first step toward “second person neuroscience” (Schilbach et al., 2013).

### Spatial, social, and imitative effects on task performance

Our two studies allow us to distinguish a number of specific effects on performance. Note that our key performance measure was the time to touch the first drum, which reflects both the planning and initial execution of the action sequence without being contaminated by differences in the movement path. In Experiment 1, participants could be primed by a VC performing a spatially (not anatomically) congruent sequence or by three balls performing a spatially congruent sequence. In this study, we found a clear spatial congruency effect (faster responses on congruent trials). When the participant's SE of the VC was included as a covariate, an effect of form emerged such that those participants who considered the VC to be more human also showed a social facilitation effect and responded faster in the presence of the VC. Social facilitation effects have been demonstrated before for VCs (Hoyt et al., 2003; Zanbaka et al., 2004). Here we further show that not all participants react toward Virtual Reality to the same extent, or show the same degree of social facilitation. Individual differences in participant's response to the VC could be caused by many different elements including their personality, their prior experience with virtual reality, and whether the VC's appearance matches themselves. The SE questionnaire used here provides useful information in interpreting our results, and it should also be included in other VR study with VCs.

Our Experiment 2 provides the core test of automatic imitation effects. In congruent trials for this version of the task, the actions of the VC were anatomically congruent with those of the participant, but not spatially congruent. This means that if participants map the VC actions onto their own body, then they will have a performance advantage for the congruent VC trials only, and show a form by congruency interaction. This effect was found, and indicates that participants can automatically imitate the VC. Note that in both Experiments 1 and 2, the action goals (tapping drum number 1, 2, or 3) are congruent for both the VC and the ball trials. The anatomical congruency effect we show here occurs over-and-above any goal congruency effects, because it is present only when the VC performs the action and not when the balls indicate the goals. It is surprising to note that adding SE as a covariate in the analysis for Experiment 2 did not help us interpret the results. This might imply that automatic imitation is not influenced by the same types of SE as social facilitation, but further studies would be needed to test this systematically.

### Future research directions

At the present stage, our sequential social congruency task implemented in virtual reality provides a new method to explore automatic imitation in a rich, more ecologically valid setting. One of the advantages of using VC in our stimuli is that in future we can easily adapt our current VR application to test other aspects of social interaction and automatic imitation. For instance, we could test the effect of in-group and out-group by changing the appearance of the VC. We can precisely manipulate the social behavior and emotion of the VC to define how different factors modulate imitation behavior (Wang and Hamilton, 2012). Future studies can also implement this task in a fully immersive virtual world (for instance, with the Oculus Rift) to facilitate the place illusion and enhance the social interaction aspect of participants' experience, and can use VR in conjunction with neuroimaging techniques such as fMRI and functional near-infrared spectroscopy. Overall, we suggest that studying social imitation behavior in rich, well-controlled virtual reality settings is a valuable method for social neuroscience with great promise for the future.

### Conflict of interest statement

The authors declare that the research was conducted in the absence of any commercial or financial relationships that could be construed as a potential conflict of interest.

## Supplementary material

Supplementary Video 1 **Participant taking turns with a VC or three balls playing a sequence of three drumming tapping**. The VC's gaze is actively tracking the participant's head movement to give a feeling of actively being watched.
