# Supplementary material for: Automatic imitation in a rich social context with virtual characters
Source: Front Psychol. 2015 Jun 9;6:790. doi: 10.3389/fpsyg.2015.00790 (PMC4460321; doi:10.3389/fpsyg.2015.00790)
Supplement: Supplementary file 2 [file DataSheet1.DOCX]

***Supplementary Material***

**Automatic imitation in a rich social context
with virtual characters**

**Xueni Pan*, Antonia F. de C. Hamilton**

Institute of Cognitive Neuroscience, University College London, London, UK

*** Correspondence:** Xueni Pan, Institute of Cognitive Neuroscience, University College London, London, UK.

s.pan@cs.ucl.ac.uk

1. **Social Evaluation Questionnaire**

**Table 1. Social Evaluation Questionnaire for interactions with Virtual Characters**

| Co-presence Questionnaire: | Not at all Very much so |
| --- | --- |
| How much did you find yourself reacting Jessie as a real person… |  |
| 1. concerning your thoughts? | 1 2 3 4 5 6 7 |
| 2. concerning your feelings and emotions? | 1 2 3 4 5 6 7 |
| 3. concerning your physical responses (e.g. gestures, facial expressions)? | 1 2 3 4 5 6 7 |
| 4. concerning your physiological responses (e.g. heart rate, sweat, blushing, etc.)? | 1 2 3 4 5 6 7 |
| Personality questionnaire: | Not at all Very much so |
| 1. I liked Jessie. | 1 2 3 4 5 6 7 |
| 2. I would like to spend more time with Jessie. | 1 2 3 4 5 6 7 |
| 3. I think Jessie is trustworthy. | 1 2 3 4 5 6 7 |
| 4. I think Jessie is sociable. | 1 2 3 4 5 6 7 |
| 5. I think Jessie is friendly. | 1 2 3 4 5 6 7 |
| 6. I think Jessie is sympathetic. | 1 2 3 4 5 6 7 |
